# Reduction in Parasympathetic Tone During Sleep in Children With Habitual Snoring

**DOI:** 10.3389/fnins.2018.00997

**Published:** 2019-01-10

**Authors:** Maria-Cecilia Lopes, Karen Spruyt, Leticia Azevedo-Soster, Agostinho Rosa, Christian Guilleminault

**Affiliations:** ^1^Institute of Psychiatry, University of São Paulo Medical School, São Paulo, Brazil; ^2^Laseeb – Evolutionary Systems and Biomed. Eng. Lab., Institute for Systems and Robotics, Instituto Superior Tecnico, University of Lisbon, Lisbon, Portugal; ^3^Lyon Neuroscience Research Center, School of Medicine, University Claude Bernard, Lyon, France; ^4^Children's Institute, University of São Paulo Medical School, São Paulo, Brazil; ^5^Sleep Disorders Clinic, Stanford University Medical Center, Stanford, CA, United States

**Keywords:** children, sleep-disordered-breathing, habitual snoring, Parasympatethic tone, heart-rate-variability, sleep, snoring

## Abstract

**Introduction:** Changes in the autonomic nervous system due to Obstructive Sleep Apnea (OSA) during the life span have been described. Some pediatric studies have shown cardiovascular effects in children who do not fit the criteria for OSA; namely children with mild sleep disordered breathing.

**Objective:** We investigated heart rate variability (HRV) during sleep in children with chronic snoring and flow limitation events during sleep.

**Methods:** Ten children and adolescents with chronic snoring and an apnea hypopnea index < 1, associated to high Respiratory Index, and 10 controls matched for age, gender, and Tanner stage were monitored following one night of habituation in the sleep laboratory. HRV was studied at each sleep stage. The time and frequency domains were calculated for each 5-min period.

**Results:** All patients were chronic heavy snorers. They presented an apnea hypopnea index = 0.8, respiratory disturbance index = 10.2/h with lowest O2 saturation 96.1 ± 2.4%. The total power of HRV was decreased in all stages (*p* < 0.05). There was also a decrease in NN50 and pNN50 during all sleep stages compared to healthy controls (*p* = 0.0003 and *p* = 0.03, respectively).

**Conclusion:** A reduction in parasympathetic tone was found in the patient group. This may represent an autonomic impairment during sleep in children with mild SDB. A reduction in HRV in children with habitual snoring could be associated with possible increases in cardiovascular risk in adulthood.

**Significance:** The study indicates that children with habitual snoring have important parasympathetic tone changes during sleep.

## Introduction

Sleep disordered breathing (SDB) in children is associated with abnormal daytime behavior (Guilleminault et al., 1982) and neurobehavioral morbidity such as behavior problems (Ali et al., 1993; Chervin et al., 2006), cognitive deficits (Blunden et al., 2000; Halbower et al., 2006), and poor academic performance (Gozal, 1998; Urschitz et al., 2003). Behavior problems also include attentional regulation, affective information processing, and behavioral and physiological flexibility (Thayer and Lane, 2000). Moreover, according to Jackman et al. (2012), there is a window of opportunity when children just have behavior changes without cognitive deficits. It has been shown that these symptoms may also occur with low apnea hypopnea index (AHI) (O'Brien et al., 2004). Snoring associated with flow limitation and increased respiratory rate during sleep in children might also be associated with similar complaints (Guilleminault et al., 2004). A sign of abnormal sleep is the increase in cyclic alternating pattern (CAP) rate in these cases, even if the AHI is lower than 1 event/h (Lopes and Guilleminault, 2006). Unlike adults, children with obstructive sleep apnea (OSA) do not usually develop high blood pressure (BP), (Guilleminault et al., 2004) although the levels of overnight urinary noradrenaline and adrenaline are increased and changes occur in the sympathetic tone that may contribute to the cardiovascular consequences of the condition (O'Driscoll et al., 2011). Children with high BP usually have a co-morbid condition such as obesity (Horne et al., 2018; Walter et al., 2018) that may lead to both abnormal breathing during sleep and even higher BP. A subgroup of normal-weight children with SDB may even present low BP (Guilleminault et al., 2004).

There has been some interest in evaluating the autonomic sympathovagal balance in subjects with OSA by heart rate variability (HRV). Investigations were previously performed in adults (Somers et al., 1995) and also in children with severe OSA (Baharav et al., 1999). The authors have been developing studies in which they investigated autonomic balance in children with snoring and low AHI (Kwok et al., 2011; Walter et al., 2013; Nisbet et al., 2014). However, upper-area resistance can be detected by **esophageal pressure** monitoring, which is an indirect measurement of **upper** airway collapse. We hypothesize that children who do not fit the criteria for OSA (AHI < 1) but have chronic snoring and flow limitation due to upper airway collapse in events during sleep can show reduced HRV.

## Methods

### Sample

Ten children (7 boys) with chronic snoring and flow limitation during nocturnal sleep between 8 and 16 years of age and 10 individually matched controls were studied.

Patients had been referred to a sleep disorders clinic for daytime complaints that varied from fatigue, tiredness, sleepiness, and reported nocturnal sleep disruption with variable difficulties in going back to sleep. Children, with parental help, responded to the “Pediatric Sleep Questionnaire” (Chervin et al., 2000). Seven days of sleep diaries indicating bedtime, nocturnal events and daytime activities were collected according to child activity (Spruyt and David Gozal, 2011). All patients and members of the control group underwent nocturnal polysomnography.

Inclusion criteria: All children whose parents had consented to their participation and agreed to the anonymous use of their polysomnographic data, exhibiting the presence of regular snoring during sleep associated with flow limitation that did not meet the criteria of hypopnea as defined by the International Classification of Sleep Disorders 3rd edition (2014), (American Academy of Sleep Medicine, 2014) but with respiratory disturbance index (RDI) >2 events/h based on nasal cannula-pressure transducer or esophageal pressure monitoring. Children were recruited during a 2-month period. These children were followed up in order to properly treat their SDB.

Exclusion criteria: Use of medication of any type in the last 3 months, presence of restless-leg syndrome or parasomnia such as night terrors, sleep walking, bruxism, as shown by interview or questionnaire, and a reported associated disorder including migraine headache in the morning. Obesity, as determined by body mass index (BMI) adjusted for ethnicity, history of premature birth, and periodic leg movement score> 5 event/hour were also criteria for exclusion.

Controls were recruited from the general community by local advertisement and word of mouth. They were age (13 ± 4 months), gender, and ethnicity matched. They underwent similar clinical and pediatric sleep questionnaire evaluation and completed sleep logs. They had no sleep complaints, normal health, and had normal sleep habits. They underwent the same polysomnography setting and scoring.

### Polysomnography

The following variables were monitored during nocturnal sleep, with lights out time based on 7 days of sleep logs: electroencephalogram (EEG) of C3/A2, C4/A1, Fz/A1-A2, O1/A2 (band pass filtered at 0.3 to 40 Hz), electrooculogram (EOG) of both eyes, chin and leg electromyograms (EMGs), electrocardiogram (ECG) with two electrodes placed laterally below the two clavicles equidistant from the sternum, and respiration, using nasal cannula pressure transducer, oral airflow (by thermocouple measurement), thoracic and abdominal expansion (with piezoelectric bands), (one out of two nights of recording included Pes monitoring, but the study night without such measurement was selected to avoid any question of possible sleep disturbance related to equipment), breath-sound intensity with a microphone (anterior neck), and arterial oxygen saturation (SaO2) via pulse oximetry (Nellcor Inc., Oakland, CA). Recordings were performed on computerized polygraphic sleep systems (Sandman™, Ottawa, ON, Canada). The sampling rate of the recorded EEG was 128 Hz with the Sandman system and 256 Hz for heart rate. Anonymized recording data were transferred to CD-ROM.

### Analysis

#### Polysomnography Analysis

All anonymized CD-ROM data were rescored for research purpose, scorers were blind to the condition of the subject. Each sleep recording was exported in the European Data Format, and the Somnologica TM (Flagra-Medcare, Reykjavík, Iceland) program was used for the HRV data. Patients were resting in a supine position during all epochs chosen for HRV analysis. In order to record sleep-stage related HRV a series of 5-min epochs were chosen in the first two cycles. In each 5-min period, ECG signals were analyzed for automatic detection of R waves. We used a minimum sampling frequency of 250 Hz for HRV analysis in accordance with the Task Force of the European Society of Cardiology (1996).

The subsequent tabulations were performed following pre-determined criteria. Sleep/wake was analyzed using the international criteria of Rechtschaffen and Kales (1968) and short EEG arousal (>3 s) according to the American Sleep Disorders Association-ASDA-arousal definition (American Sleep Disorders Association ASDA, 1992; Bonnet et al., 2007). The respiratory parameters were defined according to the American Academy of Sleep Medicine (1999, 2014) Apnea-hypopnea index (number of apnea and hypopnea per hour of sleep, AHI) was calculated. The respiratory disturbance index also included flow limitation in the nasal cannula pressure transducer recording with a decrease of at least 20% of flow associated with an increase in respiratory effort indicated by a more negative peak end inspiration in the esophageal pressure (Pes) curve, which was diagnosed as resistive breaths (Guilleminault et al., 2001).

The diagnosis of mild SDB was based on the presence of habitual snoring, clinical symptoms and the following polysomnographic criteria: apnea index = 0 per hour of sleep (/h), hypopnea index (HI) < 1/h, RDI ≤ 10/h, and oxygen saturation > 92% (Whitney et al., 1998; Stepnowsky et al., 2004).

Briefly summarized, the following variables were tabulated based on these analyses: sleep onset latency defined as three consecutive epochs of stage 1, total sleep time (TST), sleep efficiency (TST/total recording time), time awake after sleep onset (WASO), N1, N2, N3, and REM sleep stages and their percentages based on TST, arousal index per hour of sleep, RDI, and AHI.

##### Heart rate variability (HRV) analysis

This analysis focused on HRV analysis. Each record was carefully manually reviewed to exclude visual artifacts and arrhythmias before further analysis.

##### Time domain analysis

A continuous ECG recording was extracted from the obtained and cleaned recording; each QRS complex was detected, and the normal-to-normal (RR) intervals determined. Five time-domain indexes were derived: the standard deviation of all Normal to Normal intervals (SDNN); the mean of the standard deviation of the 5-min NN intervals over the entire recording (SDNN index); the root mean square of the difference between successive NN intervals (RMS) and the proportion of adjacent normal NN intervals differing by >50 ms (pNN50).

##### Frequency domain analysis

Five consecutive minutes of stable ECG, artifact-free, from N2, Slow Wave Sleep (SWS) and REM sleep periods were recorded during the second sleep cycle for N1, N2, and N3 sleep stages and the fourth sleep cycle for REM sleep were selected. Spectral indexes for HRV were computed by Fast Fourier Transforms using 5-min Hanning windows. We chose the central 5-min period of the longest above-mentioned sleep stages. The power densities in the very low frequency (VLF, 0.0033–0.04 Hz), low frequency (LF, 0.04–0.15 Hz), and high frequency (HF, 0.15–0.4 Hz) components were calculated by integrating the power spectral density in the respective frequency bands. Normalized power spectra LF/HF were also calculated. Results were expressed in ms^2^/Hz.

### Statistical Analysis

Central tendency measures were expressed as mean and standard deviation. Two-way ANOVA for repeated measures followed by a Bonferroni *post-hoc* test was used to analyze the differences in HRV and sleep stages considering two main factors: (1) SDB children and controls (group), and (2) stage N2, N3, and REM sleep (sleep stage). The level of significance for the variance analyses was set at *p* ≤ 0.05 using SPSS statistical package version 11.5. Correlations between HRV parameters and RDI were performed by means of a Spearman Correlation test.

## Results

### General Results

All patients were chronic snorers. The children's parents reported hyperactivity, irritability, impulsivity, and/or depressed mood in 8 out of 10 patients, using a non-structured questionnaire with sleep questions (see Table 1). None of them fit the criteria for OSA based on polysomnography (AHI > 1). The mean RDI and sleep parameters are outlined in Table 1. Episodes of prolonged increased respiratory effort as seen in the esophageal pressure monitoring were observed in the SDB children's group, with a Pes nadir of (−20 ± 2 cm H_2_O), but it was only considered as a respiratory event when followed by arousal. None of the control group members had abnormal sleep.

**Table 1:** Demographic and PSG data^a^.

**Subjects data**	**Patients**	**Controls**
	**(*n* = 10)**	**(*n* = 10)**
Age	12 ± 5	13 ± 4
Tanner stage	>1	>1
Behavioral complaints	8/10	1/10
Chronic Snoring	10/10	0/10
AI/h	0/h	0/h
HI/h	0.8 ± 1.1/h	0/h
RDI/h	8.1 ± 1.3/h	0/h
SaO2 nadir %	96.1 ± 2.4	96.3 ± 3.4
CAP rate %	67.2 ± 9.1	48.3 ± 5.2
Sleep efficiency %	92.5 ± 3.5	91.4 ± 5.1
Sleep stages (minutes)		
N1	9.5 ± 4.2	11 ± 6.3
N2	254± 19.4	263 ± 24.3
N3	78 ± 8.2	92.2 ± 10.2
REM	96.9± 8.4	99.1 ± 9.2
Arousal index	7.6 ± 2.1	8.5 ± 1.7

a *All respiratory indexes were calculated per hour (/ h) of total sleep time. AI, apnea index; HI, hypopnea index; RDI, Respiratory Disturbance Index; CAP, cyclic alternating pattern. None of the variables were significantly significant. Data were presented as mean ± standard deviation*.

### HRV Results

The time domain analysis showed significantly higher values of NN50 and pNN50 in the control children compared to those noted in snorers in all sleep stages. Looking at the frequency domain analysis, we found a significant decrease in total power for all sleep stages and an increase in LF/HF (a sympathetic index) for N2 sleep stage and REM sleep in the chronic snorer group. HRV results are outlined in Table 2.

**Table 2:** Two-Way ANOVA for repeated measures of HRV parameters for Snorer (*n* = 10) and Control groups (*n* = 10) according to sleep stage.

	**Snorer group N2 stage**	**Control group N2 stage**	**Snorer group SWS**	**Control group SWS**	**Snorer group REM**	**Control group REM**	***F*_(2,36)_**	***F*_(1,18)_ group**	***p***
RRi	783 ± 103	875 ± 142	785 ± 111	862 ± 159	778 ± 82	842.9 ± 118	Ns	ns	ns
SDNN	56.1 ± 23.9	88.8 ± 52.1	38.1 ± 23.7	78.8 ± 74.8	60.8 ± 29	94 ± 58.8	Ns	ns	ns
RMSSD	52.9 ± 29.4	97.1 ± 62.7	45 ± 32.5	103.6 ± 97.8	54.8 ± 45.6	89.7 ± 72.1	Ns	ns	ns
NN50	14 ± 13.1^*^	120 ± 75.4	17.3 ± 15.8^*^	115.6 ± 83.8	14.3 ± 15.1^*^	98.5 ± 63.1	Ns	19.4	0.0003
pNN50	24.3 ± 21^*^	46.8 ± 19.2	24.9 ± 23.7^*^	45.4 ± 24.2	20.1 ± 22.9^*^	35.8 ± 19.6	Ns	5.1	0.03
VLF	2436.3 ± 1235	3236 ± 4743	733.5 ± 349	1132 ± 822	3480 ± 3179	4988 ± 3777	Ns	ns	ns
LF	3181 ± 1770	3241 ± 1861	1668 ± 1157	1883 ± 944	3501 ± 1450	2455 ± 1160	Ns	ns	ns
HF	2622 ± 1247	3510 ± 1603	2903 ± 1739	3574 ± 1825	3501 ± 1450	2455 ± 1161	Ns	ns	ns
TP	5823 ± 4259^*^	9710 ± 6459	3715 ± 3074^*^	6899 ± 2591	7934 ± 5071^*^	10084 ± 5342	Ns	4	0.05
LF/HF	1.9 ± 1.3^*^	1.1 ± 0.7	0.7 ± 0.4^#^	0.6 ± 0.3^#^	2.8 ± 2.2^*^	1.1 ± 0.8	3.6	4.7	0.03, 0.04

*Data were presented as mean ± standard deviation*.

* Difference in HRV parameters in each sleep stage according to group factor;

# *Difference in HRV parameters, according to the interaction between two factors (sleep stages and groups). RRi, the interval between beat to beat (RR); SDNN, Standard deviation of NN intervals for period of interest; RMSSD, Root mean square of successive differences of NN intervals for period of interest; NN50, NN intervals > 50 ms different from previous (NN) for period of interest; pNN50, percentage of NN intervals > 50 ms different from previous (NN) for period of interest Root mean square of successive differences of NN intervals for period of interest; VLF, very low frequency; LF, low frequency; HF, high frequency; TP, total power; LF/HF, LF/HF average over 5-min periods or less that can purported to reflect sympathetic nervous system per parasympathetic nervous system balance*.

### Correlations Results

There were significant inverse correlations between the RDI and: NN50 for all sleep stages [stage 2: *r* = −0.72, *p* = 0.01; SWS: *r* = −0.62, *p* = 0.01; REM sleep: *r* = −0.51, *p* = 0.01]; pNN50 during sleep N2 sleep stage (*r* = −0.51, *p* = 0.01), and total power during SWS (*r* = −0.44, *p* = 0.04). The arousal index per hour was inversely correlated with NN50 during N2 sleep stage (*r* = −0.59, *p* = 0.01) and SWS (*r* = −0.55, *p* = 0.01).

## Discussion

We have shown previously that children without a clear decrease in oxygen saturation during sleep but with flow limitation and chronic snoring are symptomatic. Despite the absence of oxygen desaturation events, we found abnormalities in both sympathetic and parasympathetic components of the autonomic nervous system (ANS) for most sleep stages, as seen by the alteration in NN50, pNN50, Total Power, and LF/HF components of HRV. It is sometimes difficult to identify all the arousals that occur during sleep with visual scoring in children with mild SDB, but (Chervin et al., 2004), using a computerized algorithm and CAP scoring analysis, have shown that sleep disruption occurs during chronic snoring (Lopes and Guilleminault, 2006). Taken together, sleep EEG instability, chronic snoring, and increased respiratory effort impact on the ANS balance during sleep.

PNN50 and NN50 mostly reflect the parasympathetic component of the time domain HRV analysis (Bigger et al., 1989) while LF/HF reflects the sympathovagal balance to the heart estimated using the frequency domain HRV analysis. The parasympathetic component (HF) generally includes a wide range between 0.18 and 0.4 Hz. This rhythm is synchronous with the respiratory rate and mediated by the vagus nerve to the heart (Hirsch and Bishop, 1981). The total power includes the power in all frequency bands, and its reduction is generally interpreted as a reduction in HRV.

In adults and children with severe OSA and hypoxemia, an increase in sympathetic tone during sleep was found (O'Driscoll et al., 2011; Walter et al., 2018). We also observed a modest increase in sympathetic tone during N2 sleep stage and REM sleep, which did not correlate with RDI, in our population of chronic snorers, with no decrease in oxygen saturation. On the contrary, the only significant inverse correlation between RDI and ANS variables was with the parasympathetic tone and total ECG power. It has been shown that reduction in HRV in patients with heart failure and other medical conditions is associated with poor prognosis (Tsuji et al., 1996; Cohen and Benjamin, 2006).

Demonstration of abnormal regulation of the vagal tone in subjects with SDB and no repetitive decrease in oxygen saturation has already been reported in adults with Upper Airway Resistance Syndrome (Guilleminault et al., 2005), as well as the presence of low BP in children with a limited oxygen saturation decrease despite abnormal breathing during sleep (Guilleminault et al., 2004). Chronic snoring and flow limitation with a high CAP rate during sleep may abnormally change in the vagal tone as shown here. The measure of HRV was obtained in a unique situation in our study. The resting-baseline state prior to sleep could be a predictor of SDB as it enables comparation throughout the all sleep stages.

We found changes in the flexibility of cardiovascular fitness in our data, based on changes in the parasympathetic system followed by low HRV. There is a relationship between low HRV and the increase of depression and anxiety symptoms (Gorman and Sloan, 2000), and the sympathetic activation seen in anxiety disorders may represent a failure of inhibitory mechanisms as a result of the reduced parasympathetic modulation (Chalmers et al., 2014). Our results showed low HRV since childhood by chronic snoring, and it may be followed by an increased risk of cardiovascular morbidity. Moreover, the daily worry has been related with low HRV (Brosschot et al., 2007), and the measures of HRV may provide an important window into understanding stress and health (Thayer et al., 2012). The early changes in HRV from childhood into adulthood can be followed by influences in psychological and physiological self-regulation, according to a model of Neurovisceral Integration (Thayer and Lane, 2000; Thayer et al., 2009).

In 1999, Baharav et al. (1999) found a significant positive correlation between the autonomic balance, LF/HF (that estimates sympathetic tonus), and the RDI in OSA children compared to controls. However, the children described in that study had SaO2 decreases that were clearly below those seen in our group of chronic snorers where we noted a significant inverse correlation between RDI, the total ECG power, and parasympathetic tonus, suggesting predominant parasympathetic tonus impairment. We believe that one of the key differences is the degree of oxygen saturation decrease and probably also the difference in the type of abnormal breathing pattern noted with inspiration and the shortening of expiration during inspiratory snoring with a decrease in lung inflation via sympathetic activation reflex (St Croix et al., 1999). The major limitation of our study was the small size of the group we sampled using esophageal pressure monitoring. There is a need to apply new measures of sleep disruption, and HRV subtype measurement could be useful, particularly during the night because we monitor changes in sleep stages. The use of HRV is still unclear and the LF/HF ratio can be an inconclusive measurement (Billman, 2013). However, patients with insomnia may also exhibit a higher ratio of low-to-high frequency power (LF:HF-HRV), interpreted as an index of sympathovagal tone. Sleep-related changes in HRV are associated with other physiological changes (Israel et al., 2012). The evidence also suggests that HRV collected during a PSG can be useful in risk stratification models of several pathophysiological processes (Stein and Pu, 2012).

Finally, little is known about the consequences of impaired parasympathetic tonus during sleep. The most important finding in these results is the value of HRV measurement in mild sleep disordered breathing since not all sleep laboratories measure flow limitation or respiratory effort with esophageal pressure devices, thus underestimating the impact of mild sleep disordered-breathing, and describing it as habitual snoring without health consequences. The measurement of changes in HRV could be a useful tool in estimating the consequences of SDB in children and the dysregulation of the vagal tone may play a role in the reported syncope seen in late teenagers (Koenig et al., 2017) and early adult women with insomnia and UARS (Guilleminault et al., 1995; Poyares et al., 2002; Guilleminault and Davé, 2003).

## Ethics Statement

This study was carried out in accordance with the recommendations of name of guidelines, name of committee with written informed consent from all subjects. All subjects gave written informed consent in accordance with the Declaration of Helsinki. The protocol was approved by ethics commitee.

## Author Contributions

All authors have worked with the data together. M-CL: Collected the data and conducted the full review. KS: Aided in the final interpretation of the data. LA-S: Generated data from PhD thesis and contributed to the English review. AR: Introduced the topics about HRV to the authors' department and was responsible for the data together with the first author. CG: Critical role in giving advice on interpreting the data.

### Conflict of Interest Statement

The authors declare that the research was conducted in the absence of any commercial or financial relationships that could be construed as a potential conflict of interest.
